# A coordinated sequence of distinct flagellar waveforms enables a sharp flagellar turn mediated by squid sperm pH-taxis

**DOI:** 10.1038/s41598-017-13406-z

**Published:** 2017-10-11

**Authors:** Tomohiro Iida, Yoko Iwata, Tatsuma Mohri, Shoji A. Baba, Noritaka Hirohashi

**Affiliations:** 1 Oki Marine Biological Station, Education and Research Center for Biological Resources, Shimane University, 194 Kamo, Okinoshima-cho, Oki, Shimane 685-0024 Japan; 2 Atmosphere and Ocean Research Institute, University of Tokyo, Kashiwa, Japan; 3 Section of Individual Researches, National Institute for Physiological Sciences, National Institutes of Natural Sciences, 5-1 Higashiyama, Myodaiji-cho Okazaki, 444-8787 Japan; 4 Ochanomizu University, 2-2-1 Otsuka, Tokyo, 112-8610 Japan

## Abstract

Animal spermatozoa navigate by sensing ambient chemicals to reach the site of fertilization. Generally, such chemicals derive from the female reproductive organs or cells. Exceptionally, squid spermatozoa mutually release and perceive carbon dioxide to form clusters after ejaculation. We previously identified the pH-taxis by which each spermatozoon can execute a sharp turn, but how flagellar dynamics enable this movement remains unknown. Here, we show that initiation of the turn motion requires a swim down a steep proton gradient (a theoretical estimation of ≥0.025 pH/s), crossing a threshold pH value of ~5.5. Time-resolved kinematic analysis revealed that the turn sequence results from the rhythmic exercise of two flagellar motions: a stereotypical flagellar ‘bent-cane’ shape followed by asymmetric wave propagation, which enables a sharp turn in the realm of low Reynolds numbers. This turning episode is terminated by an ‘overshoot’ trajectory that differs from either straight-line motility or turning. As with bidirectional pH-taxes in some bacteria, squid spermatozoa also showed repulsion from strong acid conditions with similar flagellar kinematics as in positive pH-taxis. These findings indicate that squid spermatozoa might have a unique reorientation mechanism, which could be dissimilar to that of classical egg-guided sperm chemotaxis in other marine invertebrates.

## Introduction

Spermatozoa released from males must travel to encounter the egg. During this process, spermatozoa are often guided by chemicals from the female reproductive tract or in the vicinity of the egg^1,2^, ensuring successful fertilization. Sperm chemotaxis has long been recognized in marine organisms^3^, including marine algae^4^, cnidarians^5^, echinoderms^6,7^, and ascidians^8^. To date, studies using sea urchin models have given a comprehensive view of the cell behavioral and molecular bases for sperm chemotaxis^1,9–11^. In a shallow observation chamber, spermatozoa of the sea urchin *Arbacia punctulata* ^12^ and the ascidian *Ciona intestinalis* ^13^ swim in circular paths and exhibit drift motions along a gradient of the egg chemoattractant (i.e., resact for sea urchins and sperm-activating and -attracting factor, SAAF, for ascidians). This drift is enabled by a periodic modulation of the flagellar beat symmetry (FBS). During each cycle, spermatozoa display higher or lower FBS values when swimming up or down the gradient, respectively. In sea urchins, binding of resact to its receptor located on the flagellar membrane evokes cyclic guanosine monophosphate-mediated signaling^14–17^, which leads to calcium influx and modulates calcium-regulated events such as microtubule sliding^18–20^. This in turn leads to breaking of the FBS pattern (turning). The rate of change in intracellular Ca^2+^ ([Ca^2+^]_i_) concentrations correlates positively with the degree of curvature of a smoothened swimming path^12^. In ascidians, during the initial phase of [Ca^2+^]_i_ bursts that occur when spermatozoa swim in the field at a local minimal SAAF concentration, the flagellar waveform becomes asymmetric^13^. In both cases, [Ca^2+^]_i_ dynamics rather than absolute [Ca^2+^]_i_ concentrations play pivotal roles in sperm chemotaxis^21^.

Here, we used the spear squid, *Heterololigo bleekeri*, which produces two types of males, ‘sneakers’ and ‘consorts’, each employing a different insemination strategy to their mating partners^22^. We previously found that spermatozoa from sneaker males, but not from consort ones, exhibit pH-taxis (directional movements to acidic environments) by which spermatozoa form clusters (self-clustering) caused by local respiratory acidosis^23^. Instantaneous acidification of the sperm microenvironment could be facilitated by the action of a flagellum-located membrane-anchored carbonic anhydrase^23^, which also drives intracellular acidification with unknown mechanisms, thereby hypothesizing that chemical sensation could occur across the plasma membrane. Thus, spermatozoa can reorient their swimming direction in response to environmental pH changes. This pH-taxis of squid spermatozoa is similar to sperm chemotaxis of marine invertebrates regarding the spatiotemporal regulation of FBS in response to minute changes in the chemical microenvironment^23,24^. However, the most prominent differences are the lack of a typical transmembrane receptor (there is an enzyme instead) for a chemoattractant and the absence of any particular site to reach during pH-taxis. The former issue provides a unique gradient sensing mechanism where there is no limit for maximal receptor occupancy (see Discussion), whereas the latter raises the issue of the significance of the degree of precision control needed for directionality.

Previously, our single-cell tracking analysis revealed that sneaker spermatozoa exhibit substantially straight swimming paths while ascending a proton gradient, whereas such a straight running mode was interrupted almost exclusively by a sharp turn while descending the gradient^23^. In terms of a navigational strategy, squid spermatozoa manifest biphasic swimming modes, ‘run’ and ‘turn’, which share some similarities in behavioral aspects with bacterial chemotaxis known for a ‘run-and-tumble’ swimming regime. In a chemotactic environment, *Escherichia coli* changes the frequency of its tumbles to bias its random walk in favorable directions^25,26^. In squid species, the frequency of turn initiation also depends on swimming directions relative to a pH gradient^23^. However, under which environmental conditions spermatozoa can initiate and terminate the turning motion remains unknown in quantitative terms. Furthermore, time-resolved analysis of the flagellar motion would be expected to provide insights into how sperm cells can make a quick turn during pH-taxis. In these contexts, we tracked single sperm trajectories in conjunction with extracellular pH imaging. We also analyzed kinematics of the flagellum and sperm head using high-speed video microscopy.

## Results

### How do spermatozoa decide chemotactic turning points

**Figure 1:**
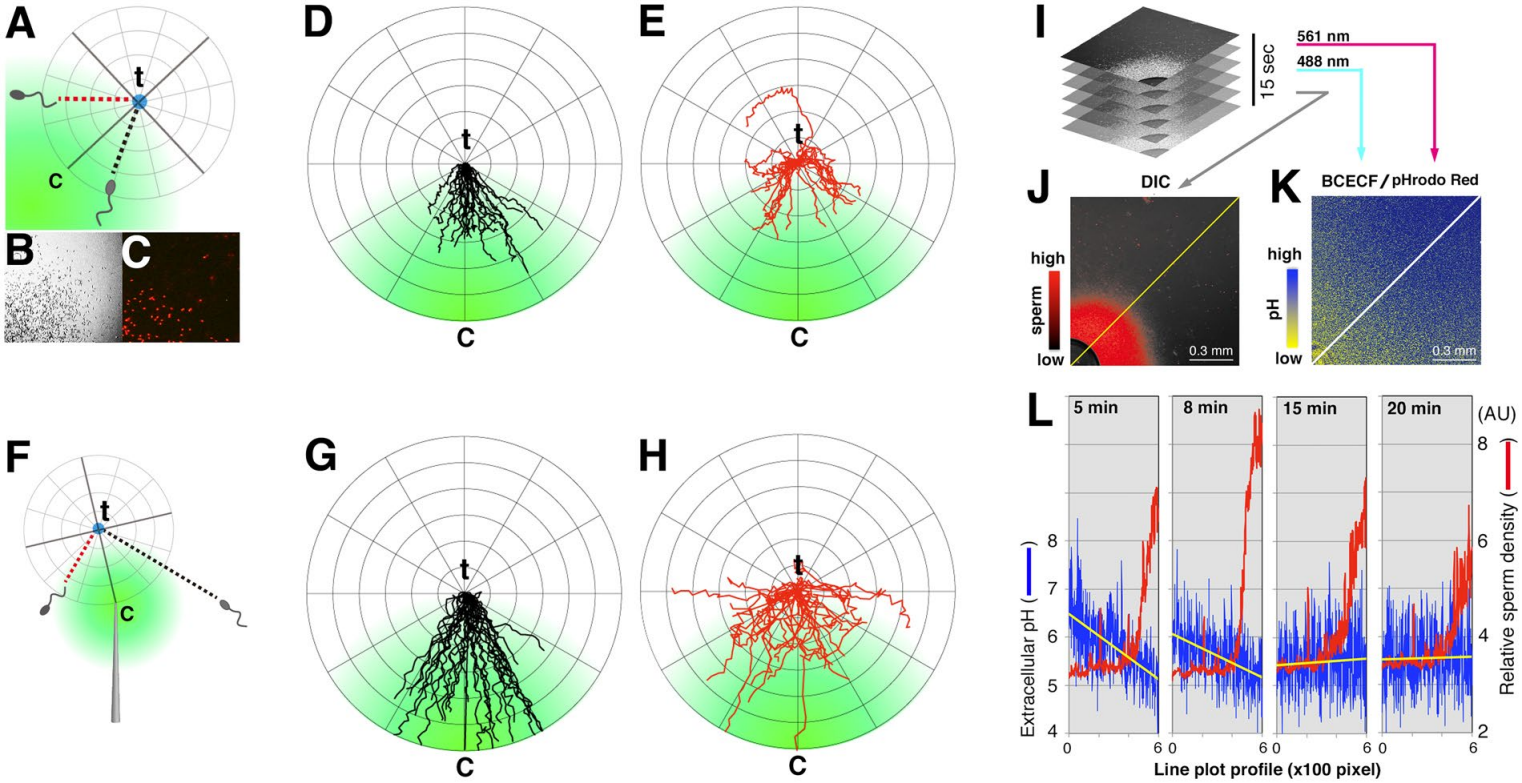
Chemotactic turning points are dictated by a combination of rate-of-change and absolute values of ambient proton concentrations. (**A**) this schematic diagram shows a turning point (t) of a sperm cell between a pair of runs near the sperm cluster boundary under a proton gradient (*green*). (**B**,**C**) approximately 1% of sperm cells were labeled with a vital stain (*red* fluorescence in **C**) to track trajectories of single cells within a cluster (**B**). (**D**,**E**) show 20 representative trajectories of straight swimmers before (**D**) and after (**E**) a short turn under a pH gradient (*green*). A circular coordinate along the t-c axis (t is set to 0, c indicates the center of the cluster) is overlaid. (**F**–**H**) the same set of experiments was carried out except that an acid-loaded pipetted was used. (**I**–**L**) determination of high-frequent turning points under a visualized pH gradient. Confocal sections scanned intermittently for 15 s (I) were merged to determine the boundary of sperm cluster (**J**). The center of the sperm cluster is located around the left-bottom corner of the panel (*black circle*). (**K**) in parallel, dynamic changes of pH were visualized by a dual pH-sensitive dye system (BCECF/pHrodo). (**L**) shown are the time-course experiments of calculated environmental pH values (*blue*) superimposed with sperm density profiles (*red*) along with the distal-proximal axis of cluster (*lines* in **J** and **K**). *Yellow lines* were obtained from linear approximation of pH plots.

It is well established that in sea urchins and ascidians, spermatozoa swim along circles or helices in two or three dimensions, respectively^27–30^. By contrast, squid spermatozoa swim in a nearly straight line with a rolling motion^23^. However, during pH-taxis, such running is interrupted by turning, suggesting that spermatozoa sense microenvironmental changes that trigger a turning motion for a suitable duration to determine the appropriate direction. To test this idea, we tracked single sperm trajectories before and after a short break of the turning episode using two different assays: self-swarming^23^ and acid-loaded pipettes. In the self-swarming assay (Fig. 1A–C), spermatozoa that swam away from a cluster almost always made a quick return around its periphery. Retrospective trajectory analysis revealed that swimming directions before a turning motion are closely aligned with a steep proton gradient (as indicated by the c–t axis in Fig. 1D), suggesting that the rate of change in extracellular pH (pH_e_) around a moving sperm cell should be greater than a critical value to trigger a turning motion (Supplementary Fig. S1). Using a hypothetical radial gradient profile and some experimental measurements, a minimal pH change required for turn initiation was estimated to be ~0.025 pH/s (Supplementary Fig. S2). By contrast, the swimming direction after reorientation was rather variable (Fig. 1E). In the acid-loaded pipette assay (Fig. 1F), these trends appeared more intensively (Fig. 1G,H). These results suggest that pH-taxis of squid spermatozoa reflects being ‘trapped’ in a cluster rather than ‘targeting’ to a particular point. Next, high-sensitive ratiometric pH_e_ imaging was carried out by the combined use of two different pH-sensitive dyes, BCECF-dextran and pHrodo Red, under confocal laser microscopy (Fig. 1I–K, see *Methods*). A pH-gradient, indicated by *blue/yellow* colorimetry (Fig. 1K), was developed radially from center of sperm mass (the lower left corner). The boundary region of sperm cluster was at ~pH 5.5 (5.5 ± 0.1, n = 4; Fig. 1L). As clusters became disassembled, the pH_e_ slope became shallower or *vice versa* (Fig. 1L). Together, these results suggest that swimming spermatozoa can trigger the turning episode only when two independent parameters—the rate of pH_e_ change and absolute pH_e_ values—meet the criteria.

### A sequence of three different flagellar waveforms governs pH-tactic reorientation

**Figure 2:**
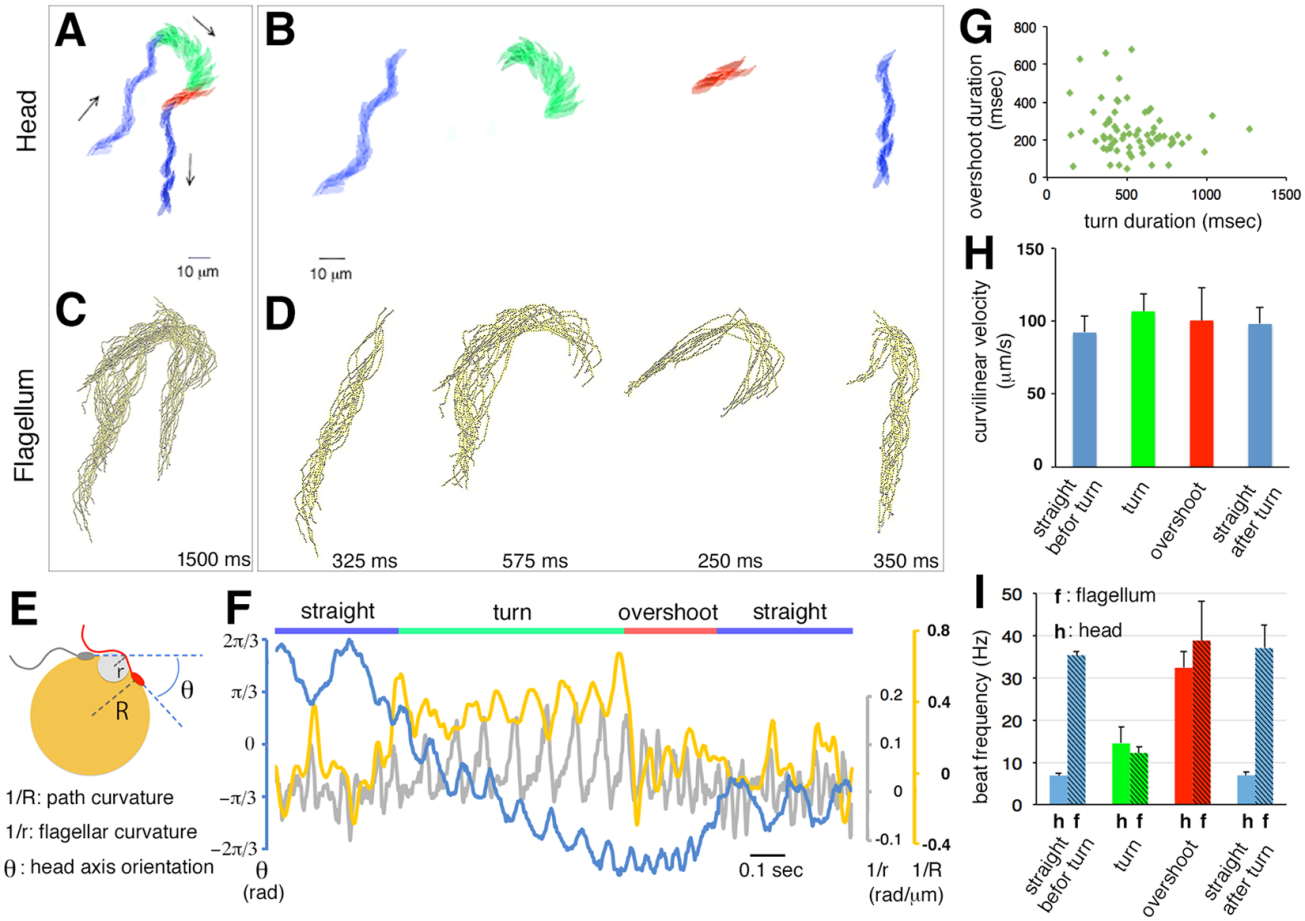
Execution of a steep turn entails a sequence of three different patterns of flagellar waveform. (**A**–**D**) representative sperm trajectories of a head (**A**,**B**) and a flagellum (**C**,**D**) during chemotactic turning were divided into four episodes (straight before turn, turn, overshoot and straight after turn). The duration (in ms) of each episode is indicated. (**E**,**F**) the kinematic parameters (path curvature, *yellow line*; flagellar curvature, *grey line*; head axis orientation, *blue line*) illustrated in (**E**) are measured and plotted (**F**) at a 2.5 ms time resolution. The flagellar curvature was measured at 5 μm from the basal end. (**G**) shown are correlation plots between the turning duration and the overshooting duration. (**H**) the curvilinear velocity of each swimming episode was measured (mean ± SEM, n = 6). (**I**) beat frequencies (Hz) of head (h) and flagellum (f) are shown (mean ± SEM, n = 30).

How do flagellar kinematics enable us to understand how squid spermatozoa make a sharp turn during pH-taxis? We applied phase-contrast high-speed video microscopy (400 fps) to analyze flagellar motion. A series of run–turn–run motions (a total of 600 frames) was extracted, and the movements of the sperm head (Fig. 2A) and its flagellum (Fig. 2C) were scrutinized separately. A pair of run motions (marked in *blue*) were clearly distinguished from a turn motion (marked in *green*) in head trajectory and bundled flagellar contours, whereas the turn episode showed curving paths in both the head and flagellum (Fig. 2B,D). Notably, immediately after the turning motion is terminated, there is a transitional state, where the head and flagellum follow straight and curved paths, respectively (Fig. 2B, *red*). Kinematic analysis (Fig. 2E) identified that such a transition state corresponds to an ‘overshoot’ motion, where a sperm head swings back from the over-turned direction (Fig. 2F, *blue* line). This motion is distinct from run or turn in terms of beat-cross frequency (BCF; the frequency at which the sperm head moves across the middle line of the smooth trajectory, Fig. 2I) and the wave propagation distance (Supplementary Fig. S3). Apparently, during the straight-run episode, BCF could be reflected by the corresponding head rotation speed rather than flagellar beat frequency (Supplementary Video S1). The duration of ‘overshoot’ motion is variable and does not correlate with the duration of turning (Fig. 2G, Supplementary Fig. S4). It should be noted that the mean head propulsion speed is constant in any mode of flagellar motion (Fig. 2H). This result suggests that the driving force works on sperm head continuously, even though the overall viscous drag that acts on entire sperm could be different between run and turn. However, time-resolved analysis identified that the flagellar beat frequency (Fig. 2F, *gray* line) became relatively low during the turning episode (Fig. 2I).

### A train of coordinated actions of steering and propulsion facilitates a sharp turn execution

**Figure 3:**
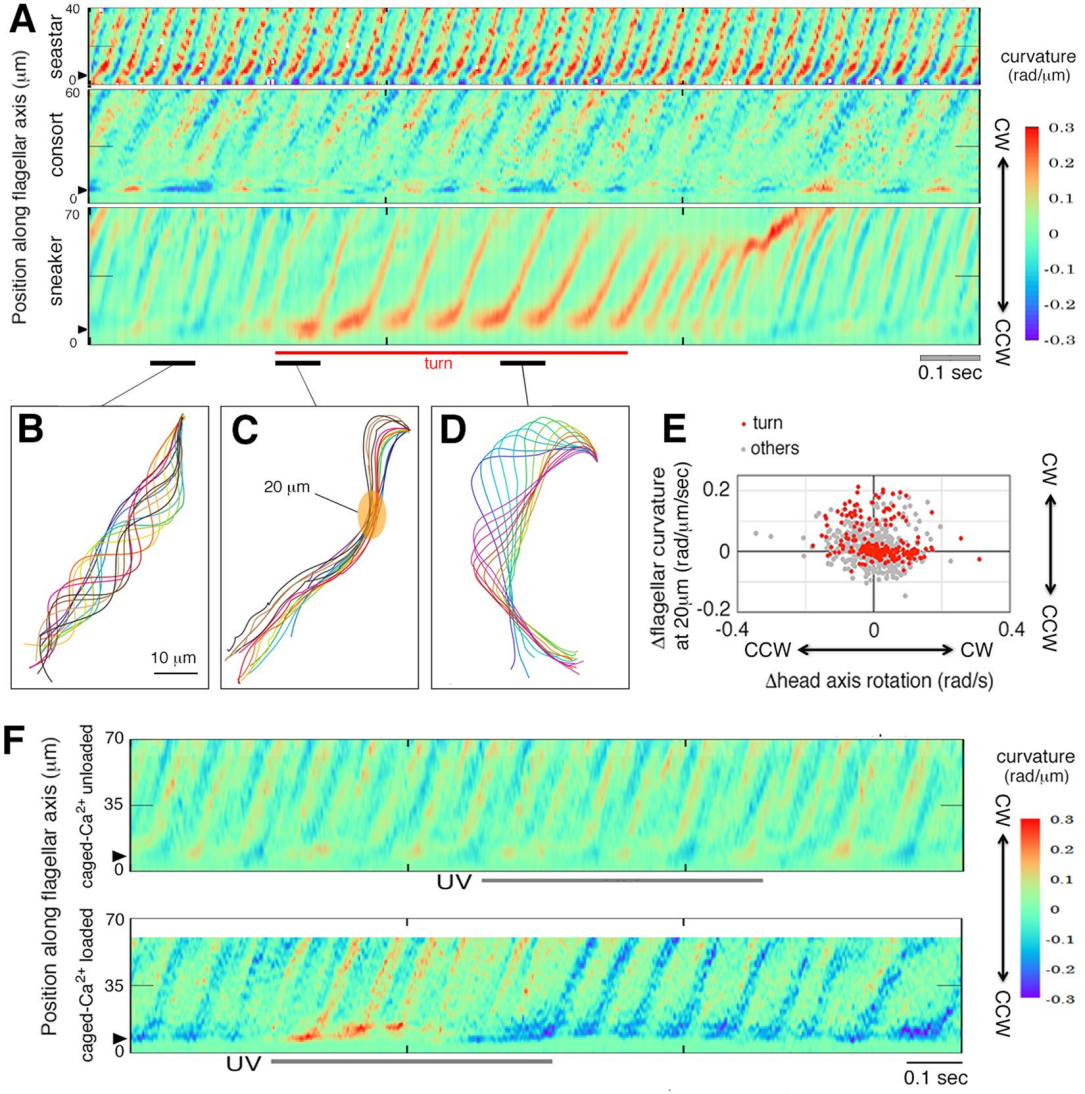
Spermatozoa steer or propel themselves independently by operating flagellar bending or bend propagation, respectively. (**A**) representative kymographs of the spatiotemporal curvature along a sperm flagellum for sea star (*top panel*), consort male squid (*middle panel*), and sneaker male squid (*bottom panel*). (**B**–**D**) typical flagellar contours for the period of straight running (**B**) bent cane-shaping (**C**) and wave propagation (*black underline*). The turning period is also marked (*red underline*). (**E**,**F**) correlation between the change in head-axis rotation and that in flagellar bending at 20 μm from the proximal end. Note that during bent cane-shaping, the initial principal bend appeared in the proximal region (>20 μm) of a flagellum. (**F**) NP-EGTA-unloaded (*top panel*) or loaded (*bottom panel*) sneaker spermatozoa were subjected to UV photolysis in the time window indicated. *CW* and *CCW* indicate the flagellar curving direction as clockwise and counterclockwise, respectively.

The above phenomena were further analyzed using the spatiotemporal dynamics of flagellar curvature along the flagellum. In sea star (Echinoderm) spermatozoa that swim in circles^31^, a pair of principal and reverse bends arise periodically and traverse the flagellum from base to tip at a constant speed (Fig. 3A, *top*). In spermatozoa of consort male squids, which are constant straight swimmers, kinematic patterns were similar to those of sea star spermatozoa with relatively lower beat frequencies and slower wave propagation speeds (Fig. 3A, *middle*). In spermatozoa of sneaker male squids (Fig. 3A, *bottom*), it was found that a chemotactic turning motion consists of a battery of ‘bent cane-shape’ formations (bending at the neck region, Fig. 3C) followed by asymmetric wave propagation (Fig. 3D) that occurs periodically until onset of the ‘overshoot’ episode. During the bent cane-shape formation (39.5 ± 3.1 ms, mean ± SEM, n = 5), the head axis rotates to a new orientation (steering) while flagellar beats are arrested; thereafter, asymmetric wave propagation pushes forward to the newly formed head axis (‘driving’; Fig. 3E).

Next, we investigated the involvement of [Ca^2+^]_I_ regulation during pH-taxis. We used a membrane-permeant caged-calcium probe to evoke a burst increment of [Ca^2+^]_i_ under a phase-contrast high-speed video microscope. In controls, an ultraviolet (UV) light flash on sneaker spermatozoa unloaded with the probe showed no signs of turning (Fig. 3F, *top*), whereas those loaded with the probe initiated cycles of bent cane-shaped quiescence and asymmetric wave propagation shortly after photolysis, causing the turn episode, and thereafter recovered to a straight swimming mode (Fig. 3F, *bottom*). These results suggest that transient calcium mobilization triggers a series of elementary motor patterns to execute the turning episode for a certain period.

### Squid spermatozoa show positive and negative pH-taxis

**Figure 4:**
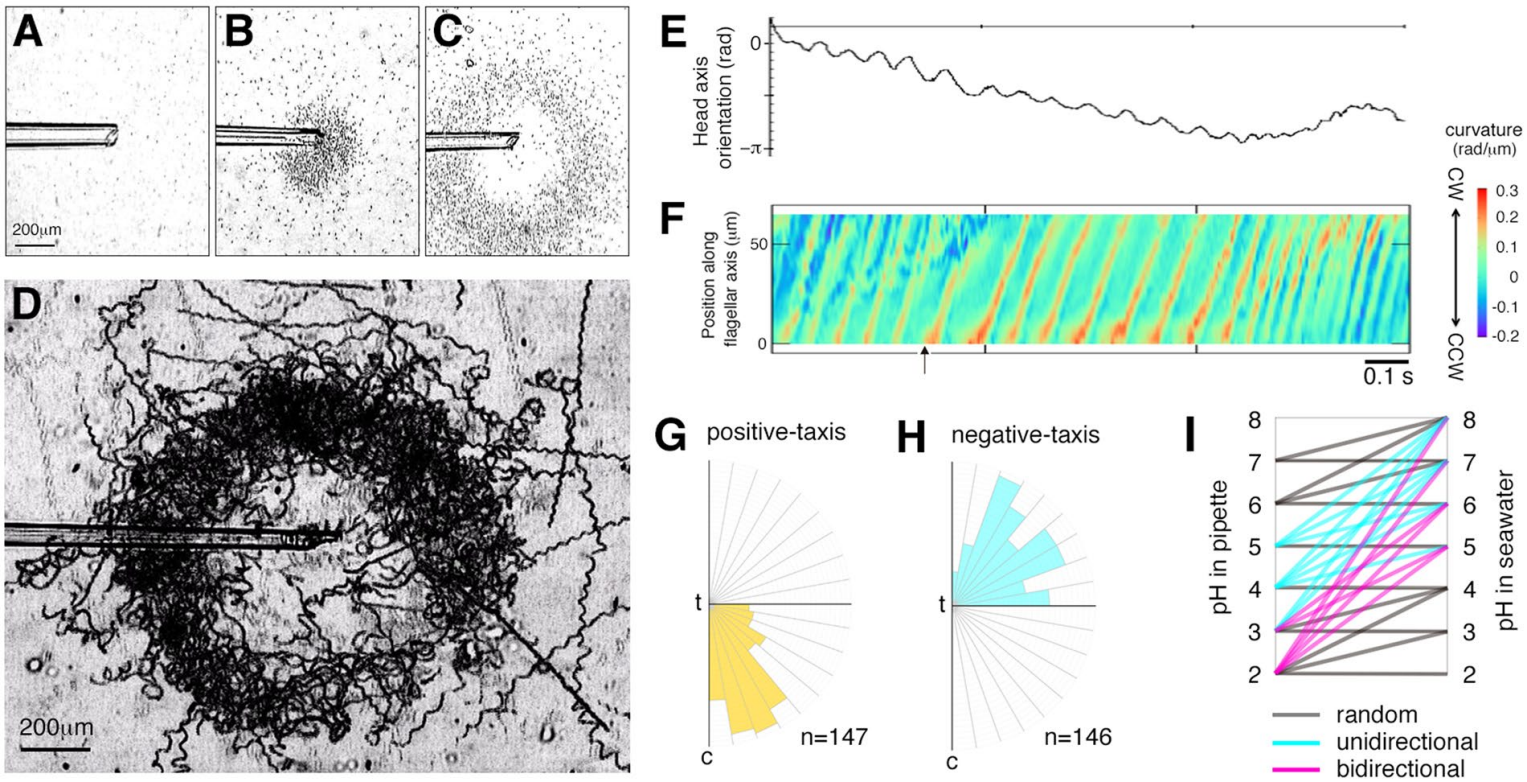
Bidirectional pH-taxis in squid spermatozoa. (**A**–**C**) the clustering behavior of spermatozoa in response to acid-loaded pipettes adjusted to pH 8 (**A**) pH 4 (**B**), and pH 2 (**C**). (**D**) trajectories (durations of 9 s) of spermatozoa in (**C**) were drawn. (**E**,**F**) a representative example is the single cell analysis of head axis orientation (**E**) and flagellar curvature (**F**) during negative pH-taxis (repelling from a strong acid microenvironment). *Arrow* indicates an initial point of the turning episode. (**G**,**H**) in (**D**), tilt angle-distributions of swimming directions after turning are scored under positive (**G**) or negative (**H**) pH-taxis. Here, c and t indicate positions of the pipette and a turning point, respectively. (**I**) types of cluster, i.e., no cluster (*random*), as shown in (**A**), simple cluster (*unidirectional*) in (**B**), and ring-shaped cluster (*bidirectional*) in (**C**), that formed in different pH gradients were determined.

It is known that some bacteria such as *E. coli* show bidirectional pH-taxis by which cells can escape from the extreme pH environments and result in accumulation in the optimal range of pH_e_^32,33^. We examined whether squid spermatozoa would possess negative pH-taxis (being repelled from strong acid microenvironments). We used a microcapillary assay where the gel was adjusted to various pH values, by which qualitative assessment for ‘pH-dependent turning capability’ is possible. As expected, sperm showed random movement (no swarming) or swarming around the microcapillary end when the gel contained citrate buffer at pH 8 or pH 4, respectively (Fig. 4A and B). However, when examined at pH 2, a ring-shaped sperm swarm was formed (Fig. 4C). Tracking analysis revealed that spermatozoa executed a turn motion at the inner edge of the ring to avoid the pipette (Fig. 4D). Flagellar turn kinematics were similar between positive and negative pH-taxis (Fig. 4E–H). Next, using this assay, various pH slopes are created by adjusting the gel and seawater at different pH values. We found that whether sperm behave either simple swarming (unidirectional) or ring-shaped swarming (bidirectional) depended on two independent parameters, i.e., absolute pH values in gel and seawater and differential pH values between gel and seawater (Fig. 4I, *cyan lines* or *magenta lines*). In particular, negative pH-taxis was observed when pH in the microcapillary pipette was adjusted at ≤3 but not at ≥4, suggesting that the threshold pH for negative pH-taxis would be between 3 and 4 (Fig. 4I).

## Discussion

Chemotactic cells commonly express cell surface receptors by which a chemical gradient is detected with a spatial sensing mechanism (e.g., measuring the difference in receptor occupancy between the anterior and posterior of a cell) for amoeboid cells^34^ or a temporal sensing mechanism (e.g., measuring the time derivative of the receptor’s occupancy) for flagellated cells^24,35–38^. In addition, chemotactic cells must tune their dynamic sensitivity range when responding to concentration ranges over several orders of magnitude^24,39,40^. For instance, bacterial chemoreceptors (e.g., CheA and CheW) adjust their sensitivity by glutamyl modifications (methylation or amidation) in the cytoplasmic domain^41^. In *Dictyostelium* cells, phosphorylation of the cyclic AMP receptor and Gip1-mediated trimetric G-protein shuttling yield proper sensitivity for gradient sensing^42,43^. In sea urchin spermatozoa, a guanylyl cyclase (GC) chemoreceptor is inactivated by autodephosphorylation that occurs rapidly after resact binding^44^. The higher the receptor occupancy, the lower the receptor affinity^17^. Although the underlying mechanism remains to be identified, it could arise from negative cooperativity of adjoining GC chemoreceptors or a negative feedback loop in the chemotactic signaling cascade^17^. However, squid spermatozoa are unlikely to adjust their sensitivity to a wide range of external pH values; instead, they set a threshold pH value or a narrow pH range; ΔpH < 1, which is equivalent with smaller than a 10-fold change in proton concentration, to trigger the FBS breaking (Fig. 1I–L). In addition, the rate of pH change in the sperm microenvironment serves as a cue for making a turning-point decision (Fig. 1A–H). The time derivative of the pH_e_ rise should be greater than 0.025/s to evoke the turning signal. In other words, if spermatozoa swim along a shallow concentration gradient of ≤0.025/s, FBS breaking seldom occurs. In bacteria, chemotactic performance is intensified in fast-swimming populations because of the increased reorientation frequency^45^. In sea urchins, the time derivative of the flagellar [Ca^2+^]_i_ changes modulates the path curvature^12^. Of particular interest, in this system, not only an increase (‘on response’), but also a decrease (‘off response’) of a chemoattractant evokes a Ca^2+^ signal in spermatozoa^11^. Thus, the rate of change in the chemical microenvironment could be tightly coupled with temporal dynamics of intracellular signaling in chemotactic cells. However, the complexity lies in the fact that squid spermatozoa show both positive and negative pH-taxis where the output flagellar responses are the same despite receiving opposing chemical changes (Fig. 4). Similar behavior is seen in *E. coli* where two major chemoreceptors, Tar and Tsr, can respond oppositely to pH changes^32,33^. In this regard, mechanism-based insight into the spermatozoon’s bidirectionality awaits identification of signal components.

In this study, the precision of chemotaxis (angular variance relative to the axis along a pH gradient) was quantified. We found that only those spermatozoa that had swum in a steep gradient (i.e., experienced fast pH_e_ changes) underwent a turning episode. By contrast, post-turning directions were more variably distributed to the source of acidity. However, this imprecise orientating system can prevent sperm dilution, so pH-taxis may have an endogenous role for preventing dispersal rather than seeking a target^46^. The behavioral difference between the squid pH-taxis and egg-guided sperm chemotaxis could also be seen in the physiological length of time during which spermatozoa may respond. During sea urchin fertilization, sperm chemotaxis can operate once in their life and only at the vicinity of the egg surface^17^, implying the working time to be less than 1 min. By contrast, squid spermatozoa continue clustering for hours after the release from the spermatophore^47^. Thus, the pH sensing system in squid spermatozoa should be persistently sustainable.

The second intriguing phenomenon is the periodicity of two different flagellar waveforms during turning. FBS breaking is initiated by a bent cane-shaped flagellar quiescence by which the head axis is rotated while the flagellar axis remains unchanged (Fig. 3C,E). This means that head reorientation occurs before the onset of asymmetric flagellar propulsion. A flagellum bends deeply at the proximal site (in the neck region) and makes a brief arrest before propagating the shear waves distally. A deep bend at the neck region causes reorientation of the head angle more effectively than that of the flagellar axis due to a relatively smaller drag force acting on the head, as compared with the 10-times longer flagellum^22^, in a viscous environment (at a low Reynolds number). Similarly, in the brown algae *Saccharina japonica* and *Fucus distichus*, biflagellate spermatozoa perform sex pheromone-dependent chemotactic U-turn movements, caused by extreme bending of the posterior flagellum^48^. In the marine bacteria *Vibrio alginolyticus* and *Pseudoalteromonas haloplanktis*, the flagellar ‘hook’ located at the base of the flagellum is mechanically unstable, which leads to buckling of the hook under hydrodynamic load, resulting in reorientation^49^. Thus, separated actions of steering and propulsion could be a conventional mechanism that facilitates such micro-swimmers to make a sharp turn^50^.

A bent cane-shaped flagellar quiescence has also been seen in sea urchin spermatozoa^51–54^, and its intrinsic factor is accounted for by a calcium overload^51^. In the squid, whether this similar behavior could also be attributed to elevated [Ca^2+^]_i_ levels remains unknown because of technical difficulties in calcium imaging^23^; however, caged-calcium experiments suggest its involvement (Fig. 3F).

Our kinematic analysis revealed that straight runs are distinguished from turns in BCF, the average path curvature (Fig. 2F,I), and more prominently, the flagellar shear angle (Supplementary Fig. S5). Such abrupt transition of the beating pattern is a hallmark in the beginning of the turn episode, whereas at the end of the turn episode, an intermediate state coined ‘overshoot’ appears prior to recovering to the straight run (Fig. 2). The turn episode consists of a repeated succession of bent cane-shaping and wave propagation; however, the repeat number is unspecified or even unrelated to the swimming direction before turning (Supplementary Fig. S4). Thus, in what exact conditions spermatozoa terminate the turning motion remain unknown. Occasionally, the turn episode continued more than a circle (>360°); nonetheless, the reoriented cells were still directed to a gradient ascent. This suggests that time for perceiving a chemical gradient to terminate the turn motion would be within the time while traveling in a circle (~2 s). Turning radius is estimated to be no greater than 21 μm for the head and 26 μm for the flagellum. For a spatial pH gradient with ΔpH 1/1.6 mm (Fig. 1L and Supplementary Fig. S2), the rate of pH_e_ change to which spermatozoa are exposed during circular or straight swimming before the turn is ≤0.016/s and ≈0.025/s, respectively. Thus, turning results in a ≥40% reduction of the rate of pH_e_ change. This estimation proves that decision for the turn termination is more difficult than that for turn initiation. Although the underlying mechanism remains unknown, it would be interesting to hypothesize that the flagellar length may affect the sensitivity of chemical sensing. Because males of the squid *H. bleekeri* produce dimorphic spermatozoa with either long (≈90 μm) or short (≈65 μm) flagellum^22^, and only long-flagellum spermatozoa exhibit pH-taxis^55^. We also found that spermatozoa of several squid species exhibit a swarming trait (due mostly to pH-taxis); however, the swarming capacity seemed different^56^. Future studies should address the precise correlation between flagellar size and the gradient-sensing capacity under a well-configured pH_e_ field.

Our current study suggests that three distinct motions, i.e., bent cane-shaping, asymmetric wave propagation, and overshooting, are executed sequentially in a coordinated fashion during pH-taxis in a shallow chamber (Fig. 2). In natural circumstances, however, spermatozoa swim in three dimensions (3D) and the directional navigation should operate in a 3D map. It is well established that swimming behaviors of spermatozoa on a glass plane are quite different from those in 3D space in mammals^57,58^ and invertebrates^11,28^. In fact, squid spermatozoa also beat the flagellum in 3D; therefore, careful interpretation is needed. Signal components should also be identified to determine commonality and specificities in comparison with egg-guided sperm chemotaxis, sour taste sensation^59–61^, or bacterial pH-taxis^32,33,62^. Nevertheless, it might be worthwhile to analyze basic flagellar dynamics of a chemotactic cell operated by different regimes from behavioral and physiological aspects.

## Methods

### Sperm preparation

Sneaker and consort male specimens of the squid *H. bleekeri* were purchased from fisheries (Aomori city, Miura city, and the Oki islands, Japan) as dead animals. Spermatophores retrieved from Needham’s sacs were stimulated (called spermatophoric reaction) to discharge a spermatangium (sperm mass in an inner tunic) from which spermatozoa were released. Male specimens of the sea star *Asterina pectinifera* were collected from the coast of the Oki islands, Japan.

### Microscopy and real-time imaging

Aliquots of 5 μl of sperm suspension at 10^4^–10^5^ cells/ml were placed between 1% bovine serum albumin-coated coverslips (22 × 22 mm) with a 16-μm width (for self-clustering assays) or a 200-μm width (for acid-loaded pipette assay). Single sperm trajectories (400 fps) were recorded using a high-speed camera (HAS-L2, DITECT Co. Ltd., Tokyo, Japan) equipped on an epifluorescence microscope (TE-2000, Nikon, Tokyo, Japan). Tracking of single sperm cells in clusters was carried out by labeling 1% of the population with 1 μm MitoTracker Red CMXRos (Thermo Fisher Scientific, Waltham, MA, USA) with a G-excitation filter set. Real-time pH_e_ imaging was carried out with a resonant scanning confocal microscope (A1R, Nikon) by scanning the peripheries of sperm clusters at a 0.5-s interval with 488/561 nm lasers and a × 10 objective lens. Two different pH indicators, BCECF (2′,7′-bis-(2-carboxyethyl)-5-(and-6)-carboxyfluorescein) and pHrodo Red (Thermo Fisher Scientific) at final concentrations of 1 μm were mixed before use, and the ratiometric values were normalized after calibration with seawaters at various pH values. The boundary of each sperm cluster was determined by overlaying 30 frames captured in succession (for 15 s). One percent agarose gel was melted in seawater acidified with HCl (pH 4.0), followed by sucking into a capillary needle. For experiments using a caged calcium probe, spermatozoa were incubated with 10 μm nitrophenyl ethylenediaminetetraacetic acid (NP-EDTA, Thermo Fisher Scientific) in seawater for 15 min at 4 °C, and thereafter replaced in chilled seawater until use in the experiments.

### Kinematic analysis

Kinematic parameters of motile spermatozoa placed in a shallow observation chamber were analyzed from high-speed video images (400 fps) with Bohboh software (http://shoji-baba.art.coocan.jp/Bohboh_V4.html). This software enables one to trace the flagellum line of each frame automatically and calculate the flagellar curvature along its axis. The path curvature and head orientation were also calculated using Bohboh from the centroid and axis of sperm heads, respectively, both traced manually. Kymographs of the spatiotemporal curvature were plotted using matrix data output from Bohboh in a 3D map on the Gnuplot graphing utility (http://www.gnuplot.info/). Sperm trajectories were obtained by means of image processing on Bohboh. The average linear velocity of sperm population (n > 100 cells) was estimated by a Sperm Motility Analysis System (DITECT Co., Ltd.).

## Electronic supplementary material

Movie S1

Supplementary information
