# Supplementary material for: A coordinated sequence of distinct flagellar waveforms enables a sharp flagellar turn mediated by squid sperm pH-taxis
Source: Sci Rep. 2017 Oct 11;7:12938. doi: 10.1038/s41598-017-13406-z (PMC5636881; doi:10.1038/s41598-017-13406-z)
Supplement: Supplementary file 2 — Supplementary information [file 41598_2017_13406_MOESM2_ESM.pdf]

Supplementary information

A coordinated sequence of distinct flagellar waveforms enables a sharp flagellar turn mediated by squid sperm pH-taxis

Tomohiro Iida[1], Yoko Iwata[2], Tatsuma Mohri[3], Shoji A. Baba[4] and Noritaka Hirohashi[1*]

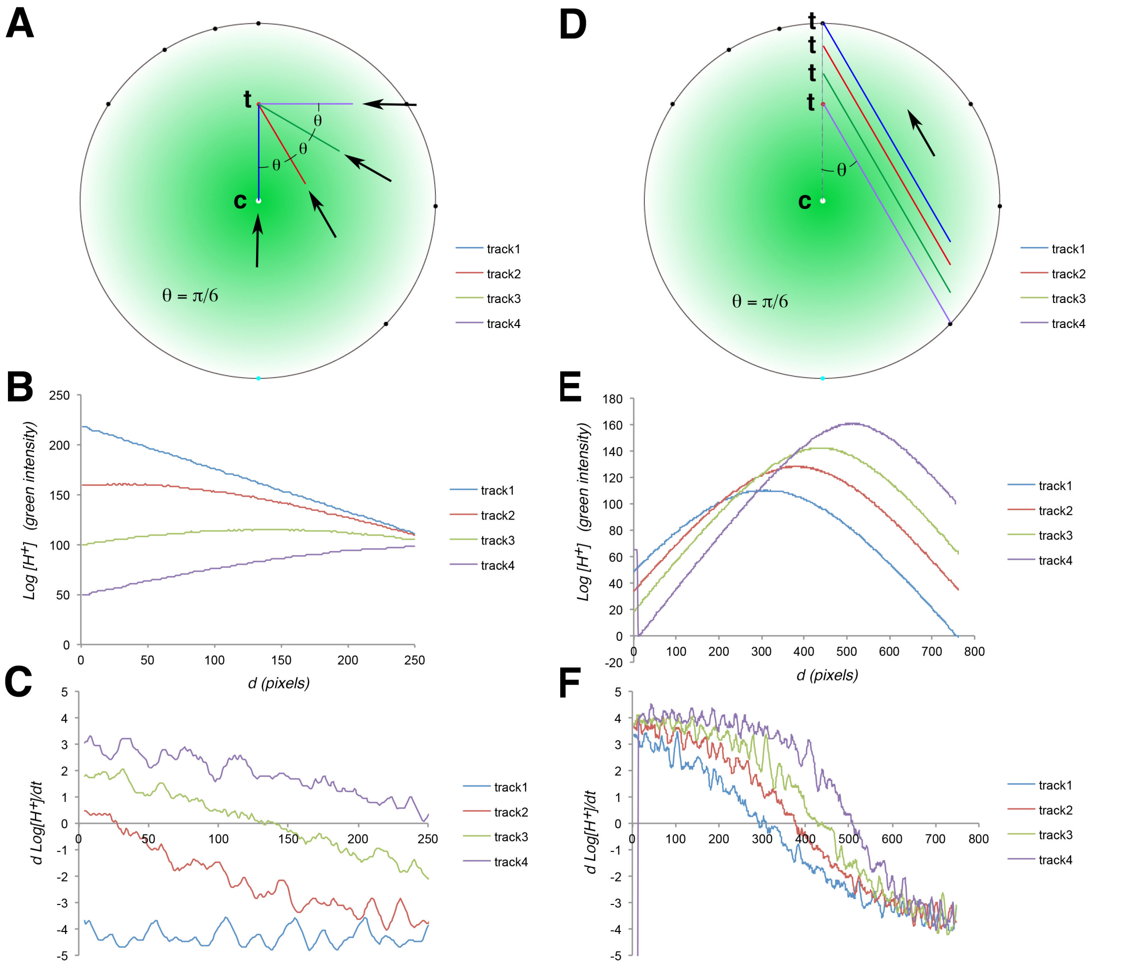

Supplementary Fig. S1. Simulation of environmental proton concentration changes near a swimming spermatozoon in a stable pH gradient. (A) This diagram shows four representative sperm tracks (colored lines) in a hypothetical linear pH gradient (*green*). Swimming directions of the sperm were indicated by *arrows*. Empirical

data (Fig. 1L) suggested that in a pH-gradient generated as the result of sperm clustering, the pH slope (d Log [H^+]/dx) was found to be considerably linear for a certain period of time (several minutes). Hence, we set a hypothetical proton gradient model, in which (c) and (t) indicate center of a radially-developed proton gradient and a turning point by a spermatozoon, respectively. Based on the results from Fig. 1D and 1G, spermatozoa that swim along track 1 (*blue* line) and track 2 (*red* line) are expected to turn at (t), whereas those that swim along track 3 (*green* line) and track 4 (*purple* line) do not. (B) Environmental changes in proton concentrations while swimming on four representative tracks were plotted as a function of travel distance from starting points (pointed by *arrows* in A). (C) Time derivatives of proton concentration changes calculated from panel (B) were then plotted. (D) Our current data presented in Fig 1D and 1G indicate that spermatozoa are able to turn only when angles (θ) between the t-c line and sperm's swimming lines were restricted to be within 30 ^{o}C ($\pi/6$). However, local turning points are fluctuated with variable distances from (c). Therefore, four potential sperm tracks (indicated by colored lines) where (t) is set at different distances from (c), but with the same (θ) were simulated. Swimming direction of all tracks was indicated by *arrow*. (E) In all tracks, sperm swim up and down the hill of proton concentrations. (F) Time derivatives of these changes revealed that all four lines are superimposed around the end of track lines (near (t) points), suggesting that despite absolute pH values are different, rates of pH change become similar around the turning points. Thus, theses analyses also support our hypothesis that rate of environmental pH change is a fundamental cue to trigger the turning episode.

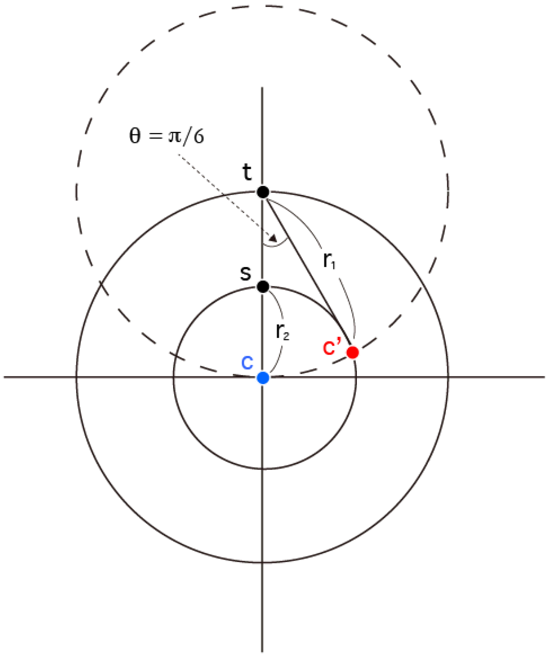

Supplementary Fig. S2. Geometric analysis for pH sensing by squid spermatozoa. As explained in Supplementary Fig. 1, spermatozoa make a turn at (t) only when swimming directions are restricted within $\pi/6$ (presented as θ) relative to the t–c axis (Fig. 1D) in a theoretical proton gradient that develops radially from the center (c) in 2-demension. First, a circular coordinate with the t-c axis can be made with a parameter (r_1): distance between t and c. Next, because θ is given at $\pi/6$, (c') will be placed as a function of (r_1). Once (c') is given, (s) where the distance s-c (i.e., r_2) equals to c'-c, is fixed.

Suppose that a proton gradient is stable at the given time period and spermatozoa commit temporal sampling while traveling from c′ to t. Then, the difference in proton concentration between c' and t equals that between s and t, where s marks a crossing-point between the c–t axis and the concentric circle with a radius of c–c′. Then, the distance between c and s (r_2) is expressed as:

$$r_2 = 2\, r_1 \sin \theta/2 \qquad (1)$$

and the distance (d) between s and t is denoted as:

$$d = r_1 - r_2 = r_1 - 2\, r_1 \sin \theta/2 \qquad (2)$$

$$d/r_1 = 1 - 2 \sin \pi/12 \approx 0.482 \qquad (3)$$

The last equation indicates that the point (s) is pined down as a midpoint of the t-c line.

Experimentally, the mean linear velocity is estimated to be 80 μm/s, and the slope of a pH gradient (i.e., this slope is equivalent to that of the t-c line) approximated to the linear function expression is estimated as 1 /1.6 mm (Fig. 1L, 8 min). Then, the rate of pH change while swimming along the c–t line is 0.05 /s. Finally, a minimal rate of the pH change required for turn initiation (i.e., along the c′–t line) would be ~ 0.025 /s.

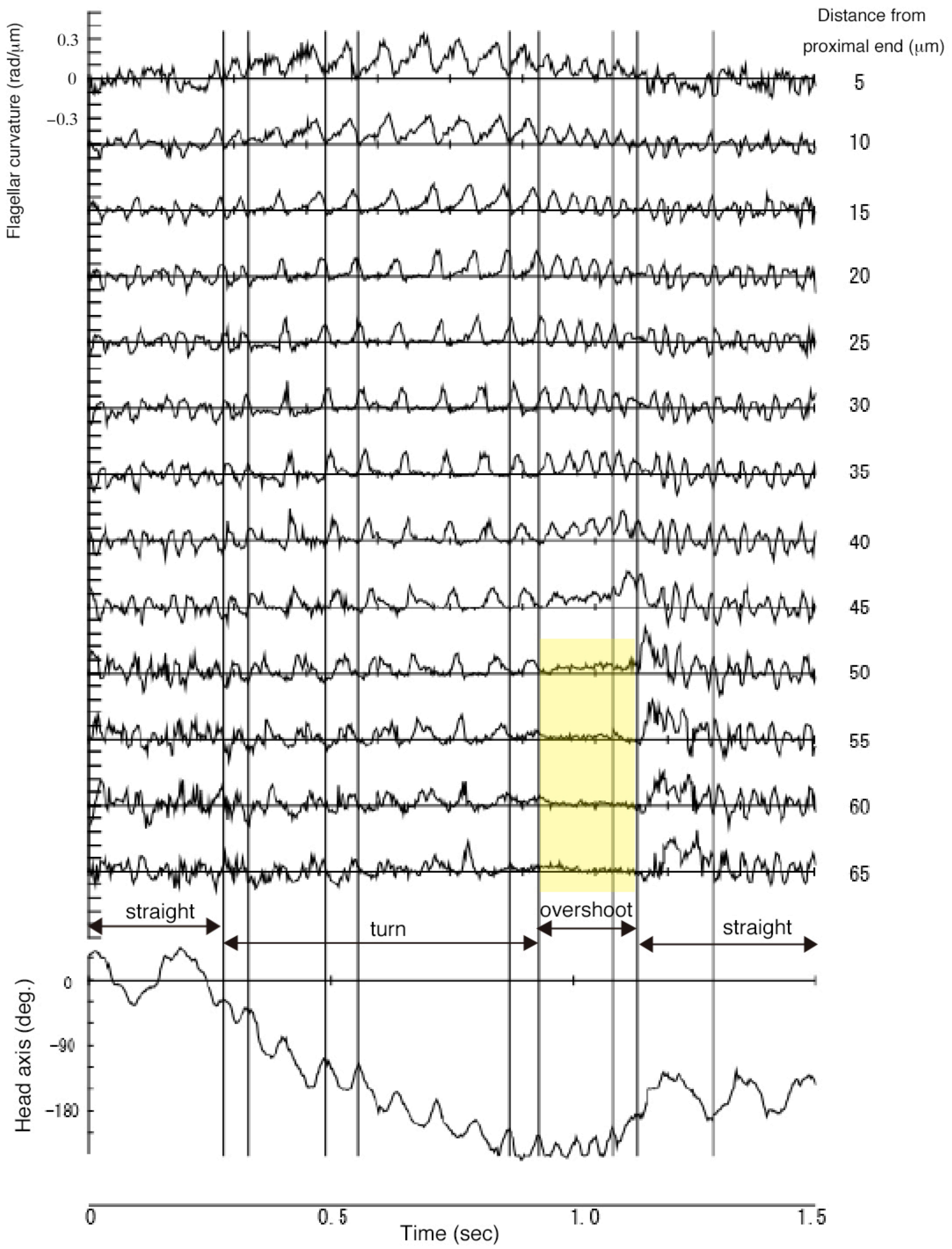

Supplementary Fig. S3. Decay in the wave propagation distance during an 'overshoot' motion. Time development of the flagellar curvature was aligned longitudinally (5-μm intervals from the proximal end). A corresponding head axis orientation is appended below. An initial head orientation (swimming toward a descending pH gradient) was set to 0. During overshooting, the flagellar wave was not transmitted to distal parts (> 50 μm) of a flagellum (in *yellow*).

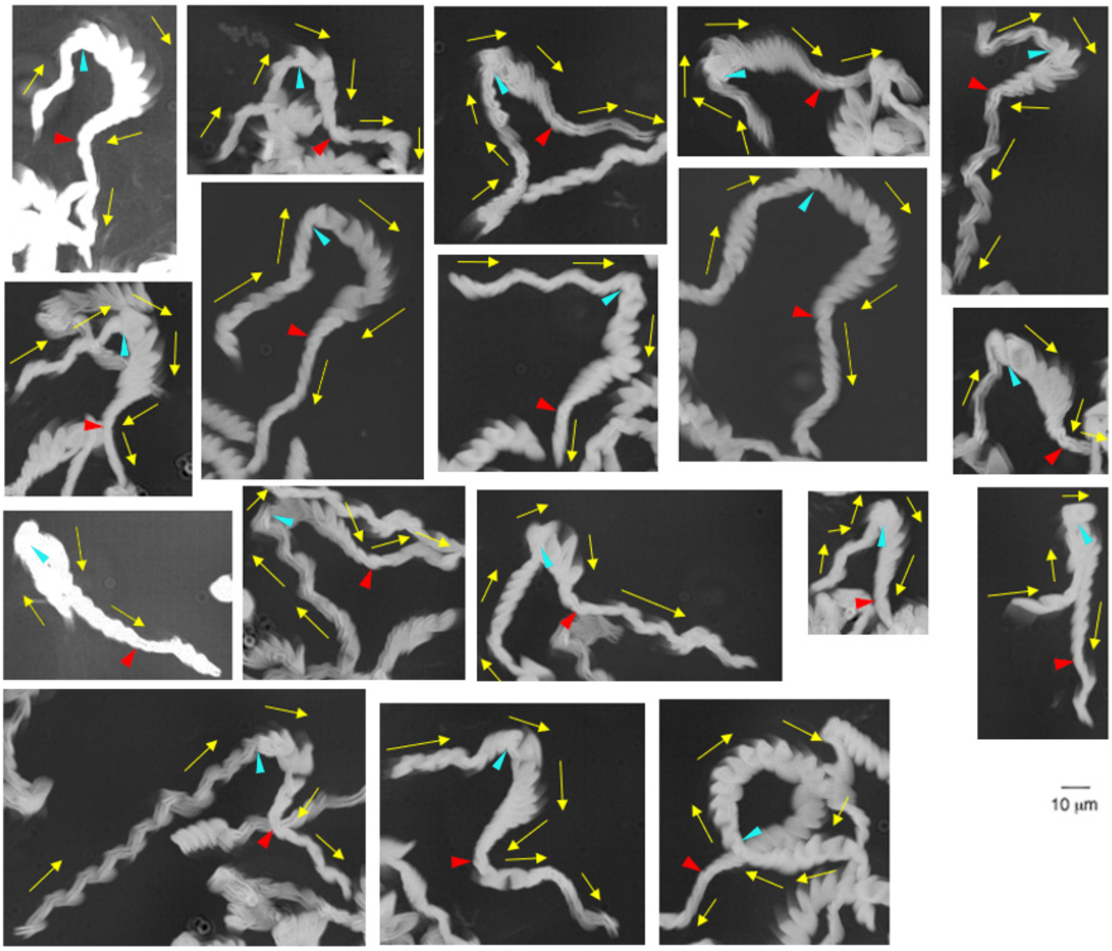

Supplementary Fig. S4. Heterogeneity in the turning duration and uniformity in the turn sequence. Head reorientation motions were traced at 2.5 ms intervals during the turn episode around the peripheries of sperm clusters. *Yellow arrows* indicate the directions of movement. *Cyan* and *red arrowheads* indicate the beginning of turning and end of overshooting, respectively. Although the turning durations and reorientation angles varied, the overshoot motion always appeared between the turn and run phases.

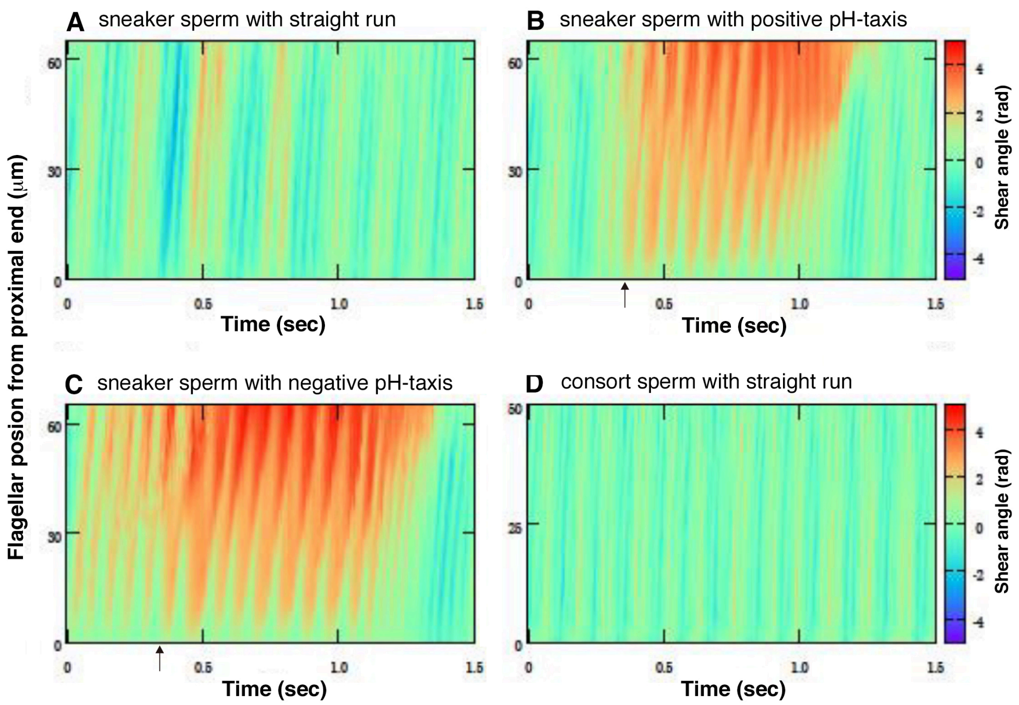

Supplementary Fig. S5. Patterns of shear angles are clearly distinguishable between straight runs and turns during pH-taxis. Shear angles were heat-mapped along a flagellum with sneaker squid spermatozoa (A–C) and consort squid ones (D) while swimming straight (A, D) or turning (B, C). *Arrows* indicate the run-to-turn transition points during positive (B) and negative (C) pH-taxis.

Supplementary Video. S1. This movie shows a slow motion playback (1/8 speed, a pulldown from 400 fps to 80 fps) of a representative spermatozoon executing a sharp turn originally captured by a high-speed camera.
